# Activity of Matrix Metalloproteinase 2 and 9 in Follicular Fluid and Seminal Plasma and Its Relation to Embryo Quality and Fertilization Rate

**Published:** 2018

**Authors:** Mojgan Atabakhsh, Iraj Khodadadi, Iraj Amiri, Hossain Mahjub, Heidar Tavilani

**Affiliations:** 1- Department of Clinical Biochemistry, School of Medicine, Hamadan University of Medical Sciences, Hamadan, Iran; 2- Endometrium and Endometriosis Research Center, Fatemieh Hospital, Hamadan University of Medical Sciences, Hamadan, Iran; 3- Social Determinants of Health Research Center and Department of Epidemiology, School of Public Health, Hamadan University of Medical Sciences, Hamadan, Iran; 4- Urology and Nephrology Research Center, Hamadan University of Medical Sciences, Hamadan, Iran

**Keywords:** Fertilization, Follicular fluid, Matrix metalloproteinase 2, Matrix metalloproteinase 9, Semen

## Abstract

**Background:**

The purpose of this study was to evaluate the activity of matrix metalloproteinase 2 (MMP-2) and 9 (MMP-9) in follicular fluid and seminal plasma and the correlation of their activities with parameters that are important in successful intracytoplasmic sperm injection (ICSI).

**Methods:**

Seventy-four infertile couples admitted to the Research Center for Endometrium and Endometriosis to carry out ICSI method were enrolled in this study. Follicular fluid was collected after retrieving the oocyte. In addition, semen samples were collected and seminal plasma was used for determination of MMP2 and MMP-9 activity. Gelatin zymography electrophoresis was applied to measure MMPs activities in follicular fluid and seminal plasma.

**Results:**

In follicular fluid, there was a positive correlation between MMP-2 activity with oocyte (r=0.27, p=0.021) or embryo quality (r=0.30, p=0.014), but no correlation was observed between MMP-2 activity and oocyte count or fertilization. Activity of MMP-9 showed positive correlation with oocyte morphology (r=0.29, p= 0.014). In addition, MMP-2 activity of seminal plasma had positive correlation with sperm count (r=0.28, p=0.015), fertilization (r=0.28, p=0.02), and embryo quality (r=0.28, r=0.026).

**Conclusion:**

MMP2 and MMP9 activities in seminal plasma have a positive effect on sperm count and motility. MMP-2 and MMP-9 activity in follicular fluid and seminal plasma could be important factors in embryo quality in patients undergoing ICSI and may affect the outcome of ICSI.

## Introduction

MPs are present in human reproduction system and are related with reproductive system functions. MMPs are classified into four main groups including gelatinizes, collagenases, stromelysin and membrane type enzymes (MT-MMPs) (1). MMPs need zinc ion for both their physiological and pathological roles. They play roles in cell migration, tissue remodeling, cell proliferation and destruction of extracellular matrix. MMP-2 and MMP-9 are members of gelatinase group and are able to degrade gelatin and collagen (2).

In male reproductive system, MMP-2 and MMP-9 are detected in seminal plasma. A previous study has shown that MMP-2 strongly affects the sperm count and also the level of MMP-2 is higher in normozoospermia rather than azoospermia. MMP-2 and MMP-9 are released from prostate and seminal vesicles; however, there is less information about the function of this enzyme in male reproduction compared to female reproductive system (3). It has also been shown that MMP-2 takes part in penetration of sperm into oocyte and functions as acrosin. Thus, probably this enzyme is located on the inner acrosomal membrane of sperm (4). MMP-2 and 9 help the movement of germ cells in spermatogenesis by digesting of extracellular matrix (5). MMP-2, 9 are involved in the breakdown of the oocyte membranes to allow sperm to enter the oocyte.

In females, degradation of extracellular matrix (ECM) is necessary for follicular development, ovulation, and luteal formation (6). Gelatinase activity has been found in the theca cell of growing follicles and the stroma of ovary (7). These enzymes help implantation by remodeling the uterus. Degradation and recycling of ECM of uterus by MMP-2 and 9 is strongly regulated. In fact, this regulation is necessary for embryo development (6). MMP-2 and MMP-9 activities increase during the later period of follicular development and these increments are along with follicular remodeling of granulosa and thecal cells (8).

In this study, the activity of MMP-2 and 9 was measured in follicular fluid and seminal plasma. Then, the correlation of activity of these enzymes with parameters that are important in successful ICSI was determined.

## Methods

Seventy-four infertile couples admitted to the Research Center for Endometrium and Endometriosis of Hamadan University of Medical Sciences to carry out ICSI method were enrolled in this study. Subjects having disorders such as endometriosis, diabetes, polycystic ovary syndrome, varicocele, breast cancer, cigarette smoking, or chronic disease were excluded from the study. Age of women and men was 23–35 and 25–40 years, respectively. All subjects were informed about the study and written informed consent was obtained from all participants. This study was approved by ethical committee of Hamadan University of Medical Sciences.

### Ovarian stimulation, oocyte retrieval and semen analysis:

All women underwent controlled ovarian hyperstimulation (COH) along with well-known GnRH agonist or antagonist protocol with recombinant FSH. When at least three follicles with 18 *mm* in diameter were detected, intramuscularly injection of human chorionic gonadotropin (hCG; 10,000 *IU*) (Choriomon, IBSA, Lugano, Switzerland) was done. Approximately 34–38 *hr* after administration of hCG and with ultrasound guidance, follicles with diameter greater than 15 *mm* were aspirated using a 17-gauge cook needle and oocytes were recovered (9). Cumulus cells of cumulus-oocyte complexes were removed from oocytes by gently pipetting with 0.1% hyaluronidase, and ICSI was performed. The fertilization was evaluated around 16–18 *hr* post-ICSI as appearance of two pronuclei and two polar bodies. The fertilization rate was calculated as the number of fertilized oocytes per number of oocytes injected. Follicular fluid was collected after retrieving the oocyte. Follicular fluid was centrifuged to exclude blood cells and impurities and stored at −80°*C* for subsequent studies. Quality and quantity of oocyte and embryo were determined by using previous published guideline (10).

The semen samples from 74 infertile men at the Research Center for Endometrium and Endometriosis were examined according to the World Health Organization (2010) laboratory guidelines (11). In the day of oocyte retrieval and after at least 72 *hr* of sexual abstinence, semen was collected during masturbation into sterile containers. Liquefaction was done at room temperature (For 30 *min*). Semen fluid were analyzed for macroscopic and microscopic parameters including sperm count, motility, morphology and viability according to the World Health Organization (WHO). After centrifugation of semen samples, seminal plasma was stored at −80°*C*.

### Zymography:

Gelatin-specific zymography technique was used for determination of MMPs activities in follicular fluid and seminal plasma. In this type of electrophoresis, polyacrylamide gel (10%) containing 0.1% gelatin (1 *mg/ml*) as substrate was used for determination of MMPs gelatinolytic activity. This method was applied two times; first, equal volume of samples (2 *μl*) was loaded to electrophoresis gel and the activity of MMPs was measured. Then, 1.5–2.5 *μl* of samples was loaded containing equal protein content. Protein content of samples was measured by Bradford assay (12). After electrophoresis, gel was incubated twice in Triton 1X, at pH=7.4 for 30 *min* and then washed by distilled water. Triton removes the SDS from gel and therefore the enzymes can be active. Then gel was incubated overnight in zymography buffer, pH=7.4 (contain Tris base aminomethane, NaCl, CaCl_2_, Na_3_N) at 37°*C*. After incubation, gel was stained with coomassie brilliant blue R250 for 30 *min* and then destained in 5% methanol, 5% glacial acetic acid. Activity of MMP-2 and MMP-9 was visualized as colorless bonds in a blue background. Gels were digitalized and activity of enzymes was quantified by Total Lap120 soft-ware (Nonlinear Dynamics Ltd, Newcastle, UK). Activity of enzymes was reported as Arbitrary Unit (AU) (13). In the zymography gel, lytic bands of MMP-2 and MMP-9 were observed. In a gel zymography, four bands can be detected, which correspond to the molecular weights of 92, 72, 62, and 82. These bands are consistent with pro-MMP -9, pro-MMP-2, MMP-2 and MMP-9 (14). Standards of gelatinases from R&D Company were also used.

### Statistical analysis:

Kolmogorov–Smirnov test indicated that the sperm count, motility, morphology, and viability were not normally distributed, but other parameters had normal distribution. Correlation between variables with normal and non-normal distribution was assessed using parametric Pearson’s coefficient (r) and non-parametric Spear-man’s coefficient (r), respectively.

## Results

### Gametes and ICSI outcomes:

The basic information (n=74) of sperm parameters were motility of 42±7 (%), count of 51±13 (10^6^/*ml*), morphology of 82±11 (%) and viability of 84±9 (%). The level of oocyte count and percentage of oocyte morphology were 8.77±6 and 71±26, respectively. Oocytes qualities were evaluated according to the growth stage and were reported as the percentage of oocytes that were in the metaphase 2 stage. The fertilization rate was 53.29±29.30, and embryo with grade A morphology was 54.53±28 in comparison to total embryo.

### MMP-2 and MMP-9 activity levels in follicular fluid and seminal plasma:

Zymography gel showed bands at 62 *kDa* and 82 *kDa* which were considered as MMP-2 and MMP-9, respectively. Levels of MMP-2 and MMP-9 activities in follicular fluid and seminal plasma are shown in table 1.

**Table 1.** Level of MMP-2 and MMP-9 activities in follicular fluid and seminal plasma of infertile couples (n=74)

	**MMP-2 activity (AU) (Equal volume)**	**MMP-2 activity (AU) (Equal protein)**	**MMP-9 activity (AU) (Equal volume)**	**MMP-9 activity (AU) (Equal protein)**
**Follicular fluid**	68.38±1.64	67.60±1.66	94.94±1.48	86.03±1.80
**Seminal plasma**	45.03±1.12	42.81±1.28	61.98±1.25	62.45±1.37

AU is an arbitrary unit that is achieved by dividing the number of pixels of MMP band curved to number of pixel of MMP standard curve

### Correlation between MMP-2 and MMP-9 activity in follicular fluid with ICSI outcome:

Correlation between MMP-2 and MMP-9 activity in follicular fluid with oocyte count, oocyte morphology, fertilization and embryo quality is shown in table 2. In follicular fluid, there was a positive correlation between MMP-2 activity with oocyte morphology and embryo quality, but no correlation was observed between MMP-2 activity with oocyte count and fertilization. Activity of MMP-9 showed positive correlation with oocyte morphology. There was not any correlation between MMP-9 activity with oocyte count, fertilization, and embryo quality.

**Table 2.** Correlation of matrix metalloproteinase2 (MMP-2) and 9 (MMP-9) activity (AU) of follicular fluid with oocyte and embryo quality (n=74)

	**Oocyte quality (%)**	**Embryo quality (%)**
**MMP-2 activity in equal volume**	r=0.26, p=0.028	r=0.3, p=0.016
**MMP-2 activity in equal protein content**	r=0.27, p=0.021	r=0.303, p=0.014
**MMP-9 activity in equal volume**	r=0.29, p=0.014	r=0.09, p=0.45
**MMP-9 activity in equal protein content**	r=0.29, p=0.015	r=0.18, p=0.14

AU is an arbitrary unit that is achieved by dividing the number of pixels of MMP band curved to number of pixel of MMP standard band curve

### Correlation of MMP-2 and MMP-9 activity in seminal plasma with semen parameters ICSI outcome:

Correlation between MMP-2 and MMP-9 activity in seminal plasma with semen parameters and fertilization is shown in table 3. MMP-2 activity in seminal plasma had positive correlation with sperm count, fertilization and embryo quality. But it did not correlate with sperm motility, sperm morphology and sperm viability. MMP-9 activity has positive correlation with sperm motility when it has been measured in equal volume, but it was not correlated with sperm count, sperm morphology and sperm viability, fertilization and embryo quality.

**Table 3.** Correlation between matrix metalloproteinase 2 (MMP-2) and 9 (MMP-9) activity (AU) in seminal plasma with sperm count, sperm motility, fertilization and embryo quality (n=74)

	**Sperm count**	**Sperm motility**	**Fertilization (%)**	**Embryo quality (%)**
**MMP-2 activity in equal volume**	r=0.28, p=0.015	r=0.10, p=0.37	r=0.24, p=0.047	r=0.28, p=0.026
**MMP-2 activity in equal protein content**	r=0.26, p=0.028	r=0.09, p=0.4	r=0.28, p=0.020	r=0.26, p=0.038
**MMP-9 activity in equal volume**	r=−0.05, p=0.36	r=0.24, p=0.040	r=0.018, p=0.88	r=0.16, p=0.19
**MMP-9 activity in equal protein content**	r=−0.03, p=0.78	r=0.193, p=0.1	r=0.07, p=0.52	r=0.149, p=0.23

AU is an arbitrary unit that is achieved by dividing number of pixels of MMP band curved to number of pixel of MMP standard curve

## Discussion

In this study, the correlation of MMP-2 and 9 activities in follicular fluid and seminal plasma with oocyte quality, sperm parameters, embryo quality and fertilization was determined. Components of oocyte growing environment are important and determination of follicular fluid components may help to predict the success probability of ICSI (15). Growth of follicles, number of oocytes, fertilization, embryo growth and implantation are factors that affect ICSI result. Ovary and specially uterus undergo major changes during follicle growth, ovulation and follicle atresia. These changes are dependent on the presence of the MMPs confirming the necessity of MMPs for these processes. Also, MMPs release growth factors from their inhibitory binding proteins, and hence increase cellular proliferative responses (16).

In our study, correlation between MMP-2 and MMP-9 activity in follicular fluid and oocyte growth showed that the increase of activity of MMP-2 and 9 results in high quality oocytes. Previous studies have shown that decreasing MMPs leads to imperfect growth of follicle or ovulation defects (17). Growth of follicles and their regression need great changes and remodeling in the ECM. Studies have shown that MMP-2 and MMP-9 increase along with the growth of follicles and in some species it is higher in larger follicles (18). MMP-2 and 9 are found in theca cells of follicles and ovary’s stroma. In animals, presence of MMPs is necessary for normal evolution of oocyte. MMP-2 plays role in different steps of follicle growing and may be a marker of good follicles. Level of MMP-9 increases when follicles grow. MMP-2 is related with follicle evolution, and MMP-9 is related with rupture of the follicles (17, 19, 20).

In a study by D’Ascenzo et al, the activity of MMP-2 and MMP-9 in follicular fluid of infertile patient undergoing *in vitro* fertilization (IVF) was lower than control group. In addition, follicular fluid MMP activity was not identified in a large number of people who had IVF. The difference of MMPs in infertile patients and control group could indicate the important role of these enzymes in IVF outcome (18).

In this study, the correlation between MMP-2 and MMP-9 activity was investigated in follicular fluid with fertilization and embryo quality. Our results showed there was not any significant correlation between MMP-2/MMP-9 activities with fertilization rate of ICSI procedure. Since for achieving fertilization in ICSI techniques, sperm through membrane around oocyte was injected, our results are predictable.

Another finding of the present study was a significant positive correlation between MMP-2 activity in follicular fluid with embryo quality. Quality of embryo is very important to get successful implantation. In ICSI, embryo must be exactly checked; the main criterion is the morphology of embryo in cleavage step (15, 21). Although follicular fluid does not exist in embryo growth environment, it probably indirectly affects through changing the oocyte quality. Our finding is in agreement with Lee et al.’s findings that reported higher activity of MMP-9 in follicular fluid in pregnant group compared to non-pregnant group. They concluded that follicular fluid of MMP-9 (But not MMP2) is necessary for successful outcome in IVF cycle (22).

There are a few studies available about the MMP-2 and 9 activities in men reproductive system (3, 23). Results of our study showed a positive correlation between MMP-2 activity in seminal plasma and fertilization and embryo quality. On the other hand, higher activity of MMP-2 of seminal plasma results in higher fertilization rate and embryo quality. In addition, it was observed that MMP-2 activity is positively correlated with sperm count, but not with other parameters. Importance of MMP activity in male fertility is indicated by some investigators. Baumgart et al. reported that concentration of MMP-2 in patients with azoospermia was lower than normozoospermic individuals (3), although information about how activity of MMP-2 in seminal plasma could affect fertilization rate and embryo quality is very limited.

## Conclusion

Our findings have shown direct correlations between activity of MMP2 and MMP9 in follicular fluid with oocyte quality and fertilization rate. MMP2 and MMP9 activities in seminal plasma have a positive effect on sperm count and motility. MMP-2 and MMP-9 activity in follicular fluid and seminal plasma could be important factors in embryo quality in patients undergoing ICSI and may affect the outcome of ICSI.

## References

[1] Curry TE Jr Osteen KG The matrix metalloproteinase system: changes, regulation, and impact throughout the ovarian and uterine reproductive cycle. Endocr Rev. 2003;24(4):428–65. 12920150 10.1210/er.2002-0005

[2] Fanjul-Fernández M Folgueras AR Cabrera S López-Otín C Matrix metalloproteinases: evolution, gene regulation and functional analysis in mouse models. Biochim Biophys Acta. 2010;1803(1):3–19. 19631700 10.1016/j.bbamcr.2009.07.004

[3] Baumgart E Lenk SV Loening SA Jung K Quantitative differences in matrix metalloproteinase (MMP)-2, but not in MMP-9, tissue inhibitor of metalloproteinase (TIMP)-1 or TIMP-2, in seminal plasma of normozoospermic and azoospermic patients. Hum Reprod. 2002;17(11):2919–23. 12407049 10.1093/humrep/17.11.2919

[4] Ferrer M Rodriguez H Zara L Yu Y Xu W Oko R MMP2 and acrosin are major proteinases associated with the inner acrosomal membrane and may cooperate in sperm penetration of the zona pellucida during fertilization. Cell Tissue Res. 2012;349(3): 881–95. 22729485 10.1007/s00441-012-1429-1 PMC3429778

[5] Chen H Fok KL Yu S Jiang J Chen Z Gui Y CD147 is required for matrix metalloproteinases-2 production and germ cell migration during spermatogenesis. Mol Hum Reprod. 2011;17(7):405–14. 21343160 10.1093/molehr/gar013

[6] Smith MF Ricke WA Bakke LJ Dow MP Smith GW Ovarian tissue remodeling: role of matrix metalloproteinases and their inhibitors. Mol Cell Endocrinol. 2002;191(1):45–56. 12044918 10.1016/s0303-7207(02)00054-0

[7] Goldman S Shalev E MMPS and TIMPS in ovarian physiology and pathophysiology. Front Biosci. 2004;1;9:2474–83. 15353300 10.2741/1409

[8] Liu D Zhu C Regulation of ovarian function by the matrix metalloproteinase system. Chin Sci Bull. 2002;47(14):1145–9.

[9] Hashemitabar M Bahmanzadeh M Mostafaie A Orazizadeh M Farimani M Nikbakht R A proteomic analysis of human follicular fluid: comparison between younger and older women with normal FSH levels. Int J Mol Sci. 2014;29;15(10):17518–40. 25268621 10.3390/ijms151017518 PMC4227176

[10] Magli MC Jones GM Lundin K van den Abbeel E Atlas of human embryology: from oocytes to preimplantation embryos. Preface. Hum Reprod. 2012;27 Suppl 1:i1. 22863770 10.1093/humrep/des229

[11] WHO WHO laboratory manual for the examination and processing of human semen. 5th ed Geneva, CH: World Health Organization 2010 287 p.

[12] Kruger NJ The Bradford method for protein quantitation. In: Walker JM, editor. Basic protein and peptide protocols. Totowa, NJ: Humana Press; 1994 p. 9–15. 10.1385/0-89603-268-X:9 7951753

[13] Ranjbaran JF Farimani M Tavilani H Ghorbani M Karimi JF Poormonsefi F Matrix metalloproteinases 2 and 9 and MMP9/NGAL complex activity in women with PCOS. Reproduction. 2016;151(4):305–11. 26733727 10.1530/REP-15-0340

[14] Buchman-Shaked O Kraiem Z Gonen Y Goldman S Presence of matrix metalloproteinases and tissue inhibitor of matrix metalloproteinase in human sperm. J Androl. 2002;23(5):702–8. 12185105

[15] Wiener-Megnazi Z Vardi L Lissak A Shnizer S Zeev Reznick A Ishai D Oxidative stress indices in follicular fluid as measured by the thermochemiluminescence assay correlate with outcome parameters in in vitro fertilization. Fertil Steril. 2004;82 Suppl 3:1171–6. 15474091 10.1016/j.fertnstert.2004.06.013

[16] Gottsch ML Van Kirk EA Murdoch WJ Role of matrix metalloproteinase 2 in the ovulatory folliculo-luteal transition of ewes. Reproduction. 2002; 124(3):347–52. 12201808 10.1530/rep.0.1240347

[17] Rienzi L Balaban B Ebner T Mandelbaum J The oocyte. Hum Reprod. 2012;27 Suppl 1:i2–21. 22811312 10.1093/humrep/des200

[18] D’Ascenzo S Giusti I Millimaggi D Marci R Tatone C Cardigno Colonna R Intrafollicular expression of matrix metalloproteinases and their inhibitors in normally ovulating women compared with patients undergoing in vitro fertilization treatment. Eur J Endocrinol. 2004;151(1):87–91. 15248826 10.1530/eje.0.1510087

[19] Horka P Malickova K Jarosova R Janatkova I Zima T Kalousova M Matrix metalloproteinases in serum and the follicular fluid of women treated by in vitro fertilization. J Assist Reprod Genet. 2012;29(11):1207–12. 23054355 10.1007/s10815-012-9853-4 PMC3510381

[20] Baka S Zourla K Malamitsi-Puchner A Makrakis E Kaparos G Demeridou S Intrafollicular levels of matrix metalloproteinases-2 and -9 in patients with polycystic ovaries are not associated with pregnancy rate during IVF cycle. In Vivo. 2009;23(1):89–92. 19368130

[21] Prados FJ Debrock S Lemmen JG Agerholm I The cleavage stage embryo. Hum Reprod. 2012;27 Suppl 1:i50–71. 22752610 10.1093/humrep/des224

[22] Lee DM Lee TK Song HB Kim CH The expression of matrix metalloproteinase-9 in human follicular fluid is associated with in vitro fertilisation pregnancy. BJOG. 2005;112(7):946–51. 15957997 10.1111/j.1471-0528.2005.00574.x

[23] Miller JE Smith TT The effect of intracytoplasmic sperm injection and semen parameters on blastocyst development in vitro. Hum Reprod. 2001;16(5):918–24. 11331638 10.1093/humrep/16.5.918

